# The impact of Inter-observation variation on radiomic features of pulmonary nodules

**DOI:** 10.3389/fonc.2025.1567028

**Published:** 2025-04-24

**Authors:** Wenchao Zhu, Fangyi Xu, Kaihua Lou, Xia Qiu, Dingping Huang, Shaoyu Huang, Dong Xie, Hongjie Hu

**Affiliations:** ^1^ Department of Radiology, Sir Run Run Shaw Hospital, Zhejiang University School of Medicine, Hangzhou, China; ^2^ Medical Imaging International Scientific and Technological Cooperation Base of Zhejiang province, Hangzhou, China

**Keywords:** inter-observation variation (IOV), nodule segmentation, radiomics, overall concordance correlation coefficient (OCCC), pulmonary nodules

## Abstract

**Objective:**

In this study, we aimed to comprehensively and systematically analyze the radiomic features of pulmonary nodules and explore the influence of inter-observation variation (IOV) in segmentation regions of interest (ROI) on radiomic features, providing reference information for pulmonary nodule radiomics research.

**Method:**

Six clinicians with varying experience and expertise manually outlined ROIs for 232 pulmonary nodules, while an artificial intelligence (AI) algorithm was trained for automated segmentation. The segmentation by the most experienced cardiothoracic diagnostician (Doctor A) served as the reference standard. Inter-observer variability was assessed through diameter measurements, segmentation ROI consistency analysis, and radiomic features stability analysis.

**Results:**

Of all radiomics features analyzed, 1071 (85.96%) demonstrated good stability (overall concordance correlation coefficient [OCCC] ≥ 0.75), with 766 (61.48%) exhibiting very good stability (OCCC ≥ 0.90). Among the eight radiomic feature types, Original _first-order, Original_GLCM, Original_GLRLM, Original_GLSZM, LOG, and wavelet features all achieved stability rates exceeding 80.00%, with 91.59% of the LOG features having good stability. The Original features showed good stability (median OCCC: 0.92-0.95, IQR: 0.12-0.19), both in the overall distribution and in the different feature categories. The median OCCC value for the LOG features (median: 0.94, IQR: 0.08) was significantly higher than that for the Wavelet features (median: 0.91, IQR: 0.13). There was no statistically significant difference in stability between the Original and LOG feature subgroups (P > 0.05). In contrast, statistically significant differences were observed between the wavelet feature subgroups (P < 0.05), with Wavelet_LLL and Wavelet_LLH transformation yielding higher stability.

**Conclusion:**

Segmentation results indicated that while IOV influenced radiomic features of pulmonary nodules, most (85.96%) of the features were well stabilized and relatively unaffected. Enhancing segmentation ROI consistency helps minimize the impact of IOV on the radiomic features of pulmonary nodule images. Original and LOG features demonstrated high stability, whereas Wavelet features were more susceptible to IOV.

## 1 Introduction

Radiomics, introduced by Lambin et al. in 2012, has revolutionized the scientific and clinical applications of medical imaging by offering new perspectives for research and practice (1, 2). It employs advanced computer algorithms to process high-dimensional images, extract numerous parameters undetectable by traditional methods, and provide comprehensive insights for diagnosing and managing clinical diseases (3–7). Radiomics has shown significant promise in pulmonary nodule research, particularly in malignancy discrimination, histopathological classification, and prognostic prediction (5, 8, 9).

Liu et al. analyzed imaging data from 875 patients with pathologically confirmed pulmonary nodules, utilized the Least Absolute Shrinkage and Selection Operator for feature screening, and constructed a benign/malignant differentiation model based on 20 radiomic features. This model demonstrated superior performance in the validation group cohort (area under the curve: 0.809; 95% confidence interval (CI): 0.745–0.872) (10). Another study explored the application of radiomics in predicting the invasiveness of lung adenocarcinoma presenting as sub-centimeter ground-glass nodules, finding a strong correlation between the radiomic signature and invasiveness (P <0.0001) (11). Despite the growing body of literature on radiomic and lung nodules, challenges such as model robustness persist (12), and significant variability exists in the imaging histologic features identified across studies (5, 13, 14).

The radiomics workflow comprises four key steps: data collection, data segmentation, feature selection and model construction, and validation. Among these, data segmentation, which involves delineating the region of interest (ROI) in the original images for subsequent feature extraction, is crucial and challenging. Segmentation methods include manual, semi-automatic, and automatic approaches. Currently, pulmonary nodule radiomics studies rely on manual or semi-automatic outlining involving human-computer collaboration to obtain ROIs. However, manual outlining is both time-consuming and laborious, with the subjectivity of operators introducing inter-observation variation (IOV) in ROI delineation (15–17). This variability in segmentation can alter radiomic feature values, impacting the results of radiomics analyses.

Initial investigations have explored the relationship between IOV and radiomic features (13, 15, 17). For example, Leo et al. evaluated the effect of ROI segmentation variability on radiomic features in non-small cell lung cancer (NSCLC), head and neck squamous cell carcinoma, and malignant pleural mesothelioma. Their findings revealed that the stability of radiomic features was significantly correlated with the DICE coefficient (DC) of ROI segmentation and that feature stability varied between tumor types (18). Similarly, Haarburger et al. compared radiomic features extracted using manual and algorithmic segmentation of the lesions of the lungs, liver, and kidneys. Their results indicated that shape and first-order features demonstrated the highest stability despite segmentation ROI transformations (19).

In summary, IOV in ROI segmentation affects radiomic features. Although radiomics have been widely applied to pulmonary nodule research, studies specifically addressing the impact of IOV on the radiomic features of pulmonary nodules remain limited. The small sample size and heterogeneity of study populations in these investigations further contribute to the lack of objective and reliable conclusions. A comprehensive and systematic analysis of IOV and its influence on radiomic features in pulmonary nodule segmentation is therefore crucial. We aimed to systematically analyze the radiomic features of pulmonary nodules, evaluate the impact of IOV in ROI segmentation, and provide reference information to advance radiomic research in pulmonary nodules.

## 2 Materials and methods

### 2.1 Research object

This study retrospectively analyzed medical records of patients who visited Sir Run Run Shaw Hospital, Zhejiang University School of Medicine, for pulmonary nodules and underwent chest computed tomography (CT) in the radiology department between July 2021 to August 2021. The CT images were retrieved from the Picture Archiving and Communication System in the Radiology Department on a case-by-case basis.

Case inclusion criteria:

[1] Complete clinical data available for the study at our hospital.
[2] Chest CT performed at our hospital.
Case exclusion criteria:
[1] Lesions larger than 3 cm in diameter.
[2] Absence of nodular lesions.
[3] Poor quality CT images affecting lesion assessment.
[4] Lack of thin-layer lung window CT images.

**Figure 1:**
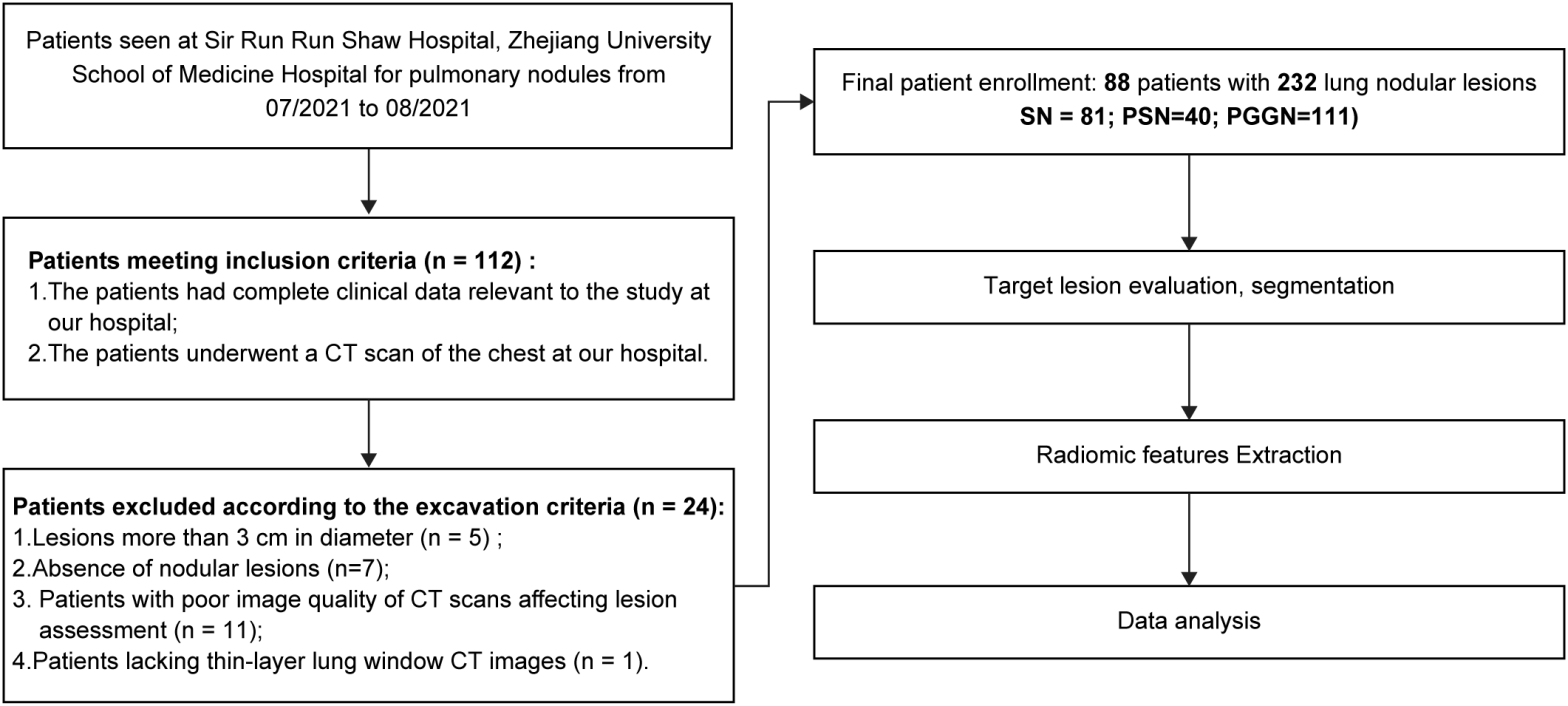
Schematic diagram of case screening and study flow.

Between July 2021 to August 2021, 112 patients met the inclusion criteria. Among these, five patients had lesions exceeding 3 cm in diameter, seven lacked identifiable nodular lesions on CT images, 11 had poor-quality CT images due to respiratory artifacts or other issues, and one patient lacked thin lung window images with a 1-mm layer thickness. Based on the above nadir criteria, 88 patients (28 males, 60 females; age range: 27–83 years; median age, 56 years) with 232 pulmonary nodular lesions were enrolled in this study. Among them, 49 patients (14 males, and 35 females) had multiple nodules. The patient screening and experimental procedures are shown in **Figure 1**.

### 2.2 Image acquisition

To exclude the influence of variations in scanning equipment, protocols, and reconstruction parameters, all patients enrolled in this study underwent chest CT scanning using the Siemens SOMATOM Definition Flash scanner in the Department of Radiology, Shaw Hospital, Zhejiang University School of Medicine. The scanning parameters were as follows: tube voltage of 120 KV, automatic tube current modulation, rotation time of 0.5 s, scanning matrix of 512 × 512, and image reconstruction layer thickness of 1.0 mm. Detailed scanning parameters are summarized in **Table 1**. Thoracic spiral CT scanning was performed from the lung apices to the lung base bilaterally. Before the procedures, patients received a detailed explanation of the examination process, including instructions on the precautions to be taken during the examination. They were trained to exhale and hold their breath during the scan. During the examination, patients were instructed to listen to the doctor’s request, inhale deeply, and hold their breath to complete the scan. The acquired images were reconstructed using a standard algorithm in the lung window, and multi-planar reconstruction was performed on a postprocessing workstation.

**Table 1:** Check device and scanning parameters.

CT Model number	Siemens Somatom Definition Flash
Reconstruction layer thickness (mm)	1
Tube voltage (KV)	120
Tube current (mAs)	Smart
Rotation time (s)	0.5
Scanning matrix	512*512

### 2.3 Image analysis

Six medical practitioners with varying specialties and work experience (Doctors A–F) were invited for training, followed by a blinded assessment of the same pulmonary nodule data. The participants’ expertise ranged as follows: Doctor A was a cardiothoracic diagnostician with 14 years of experience, Doctor B was a non-cardiothoracic diagnostician with 10 years of experience, Doctor C was a cardiothoracic diagnostician with 8 years of experience, Doctors D and E were diagnosticians with 3 years of experience, and Doctor F was a non-radiology clinician with 3 years of experience.

#### 2.3.1 Nodule assessment

##### 2.3.1.1 Consensus evaluation

Each target nodule was evaluated on a diagnostic radiology computer screen by three senior diagnostic imaging doctors (Doctors A, B, and C). In cases of disagreement, the three doctors deliberated to reach a consensus.

##### 2.3.1.2 Type of nodule

Based on nodule density in the CT images, pulmonary nodules were classified into three categories: solid nodules (SN), partially solid nodules (pSN), and pure ground-glass nodules (pGGN). pSN may also be referred to as mixed ground-glass nodules (mGGN) (20–22).

##### 2.3.1.2 Location of nodule

Lung tissue was divided into five lobes according to the interlobular pleural alignment: left-up lobe, left-down lobe, right-up lobe, right-middle lobe, and right-down lobes. Pulmonary nodules were classified by location into central and peripheral pulmonary nodules (23, 24). Central-type pulmonary nodules were defined as those occurring in lung segments or bronchial locations above the lung segments, while peripheral-type pulmonary nodules occurred in bronchial locations below the lung segments (23, 24).

##### 2.3.1.3 Diameter measurement

Three senior diagnostic imaging doctors, blinded to the patients’ clinical information, independently reviewed the CT images on a specialized diagnostic computer screen in the radiology department. Each Doctor individually measured the diameter in centimeters, which is the most commonly used quantitative characteristic of nodules. The nodule diameter was measured using the standardized long and short diameter method: the maximum long diameter and the maximum short diameter, perpendicular to the largest dimension of the nodule, were measured, and their mean was taken as the nodule diameter. The average of the measurement from the three senior diagnostic imaging doctors was recorded as the final nodule diameter for this study.

#### 2.3.2 Nodule segmentation

##### 2.3.2.1 Manual segmentation

Sweep thin-layer CT images of 232 pulmonary nodules included in this study were loaded into the open-source image processing software ITK-SNAP (www.itksnap.org) for image segmentation (25). Six doctors performed precise contouring along the boundaries of the nodule manually, ensuring that they avoided including blood vessels, bronchial tubes, and adjacent structures. In this study, original DICOM images were first imported into ITK-SNAP. Then, the six doctors could zoom in and adjust the window width and level to clearly observe the lesion, ensuring precise region of interest (ROI) delineation.

##### 2.3.2.2 Fully automatic segmentation

**Figure 2:**
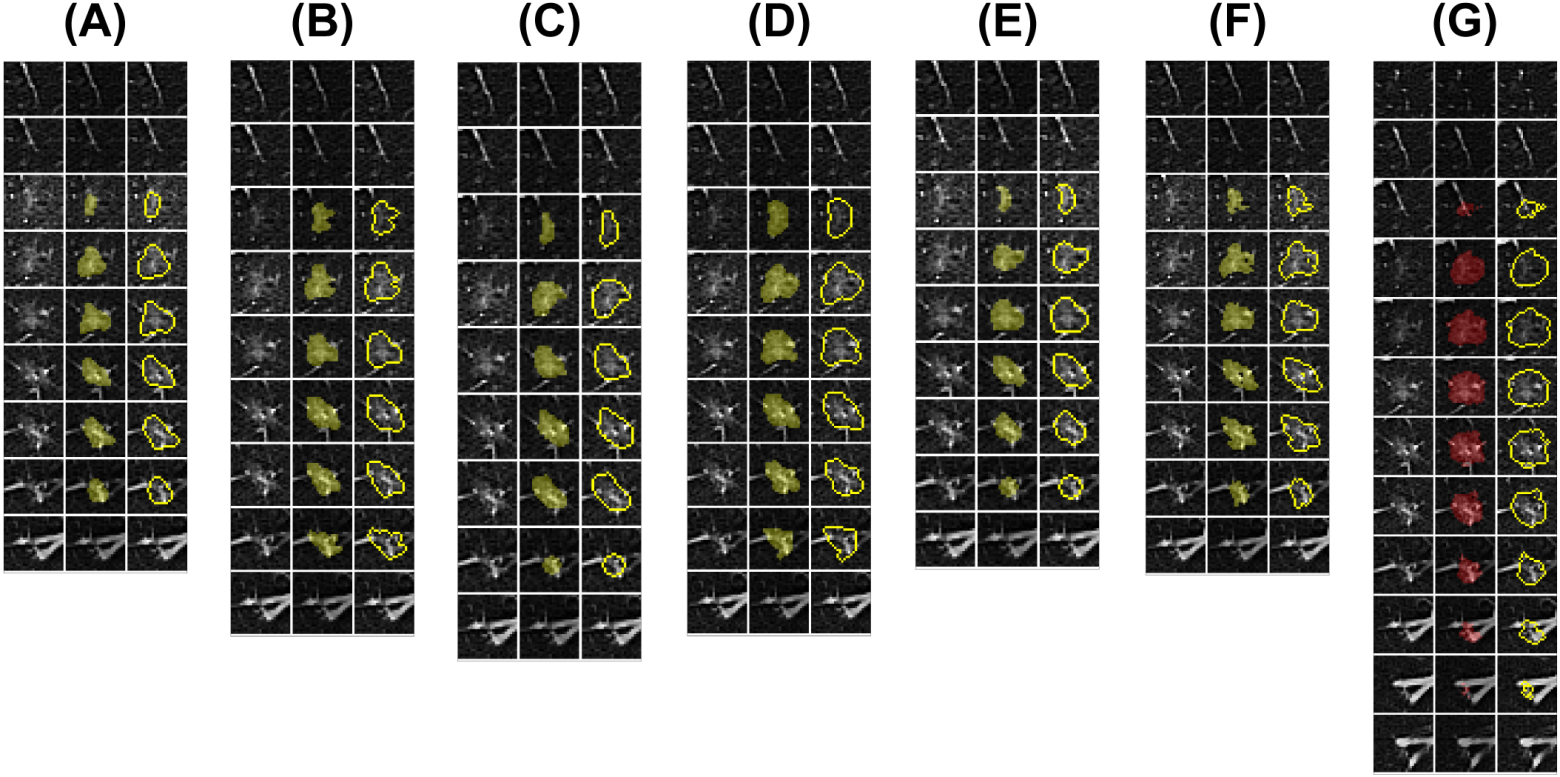
Doctors’ and AI algorithm’s lesion segmentation results. Six medical practitioners were invited for training (Doctors A-F), after which the same pulmonary nodule data were blindly evaluated for outlining, and deep learning algorithms were synchronously trained for fully automated segmentation. **(A–F)** Segmentation ROIs of Doctors A-F; **(G)** Segmentation ROIs of the AI algorithm. The subfigure displays the CT images with pulmonary nodule levels and paired ROIs from left to right.

In this study, an AI algorithm was synchronously trained to automate the segmentation of the target pulmonary nodules (**Figure 2**). Considering that most advanced medical image segmentation networks use U-Net as the backbone network and that pulmonary nodule segmentation involves three images, this study adopted the standard 3D U-Net network as the baseline and introduced improvements.

**Figure 3:**
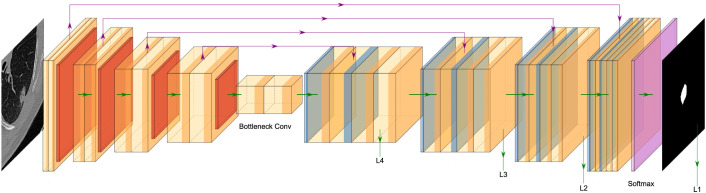
Improved 3D U-Net network structure.

The Improved 3D U-Net network structure (**Figure 3**) consists of two main components: the encoder and the decoder. The encoder consists of a cascade of five convolutional blocks. In each convolutional layer, the feature map undergoes a maximum pooling operation, halving its size. The decoder comprises four cascaded inverse convolution blocks, where the feature map doubles during each inverse convolution operation. The feature maps from corresponding layers of the encoder are jump-connected to the decoder, enabling splicing to enrich decoder features. The number of channels is adjusted using convolutional layers with a convolutional kernel size of 1×1. Finally, the decoded feature maps of the decoder are subjected to a convolution and softmax operation to generate a final probabilistic prediction map with two output channels. To enhance the richness of the encoded feature information, improve segmentation accuracy and enrich the features, attention mechanism convolution modules were integrated after each convolutional layer of the network. Additionally, deep supervision was incorporated at the side output of each layer of the decoder ( ${\text{L}}_{2},{\text{L}}_{3},{\text{L}}_{4}$ ). This approach addresses the issues of deep neural network training gradient disappearance and slow convergence speed while also providing regularization to further improve segmentation performance.

The pulmonary nodule training data used for the improved 3D U-Net network were sourced from the LIDC-IDRI public dataset (https://www.cancerimagingarchive.net). This dataset includes chest medical image files (e.g., CT, X-ray) and the corresponding lesion annotation information of diagnostic results. The information was collected under the auspices of the National Cancer Institute. Before training the network, pulmonary nodule sites were extracted from the dataset. The extraction process involved selecting a 3D CT image of size 64*64*32 centered on the pulmonary nodule based on the provided annotations. The gray values of the images were normalized to the range of 0–1, before being input into the network for training.

#### 2.3.3 Radiomic features extraction

This study utilized the Pyradiomics package to analyze all nodal segmentation data and extract radiomic features (26). Before feature extraction, segmentation data were normalized to minimize the impact of confounding factors.

A total of 1,246 radiomics features spanning eight categories were extracted for each nodule, as detailed in **Table 2**. Based on the computational principle, the extracted radiomic features can be broadly classified into four major categories (26–28): [1] First-order features, which describe the voxel intensity distribution within the ROI using basic statistical metrics, such as skewness, kurtosis, and variance. [2] Shape features, which describe the two-dimensional and three-dimensional dimensions and shape of the ROI, including diameter, volume, and surface area. [3] Textural features: describes the grayscale distribution patterns and structural arrangements within the ROI. The computation of texture features is more complex compared to first-order and shape features (29). Texture features are second-order features that are not directly extracted from the image. They are first extracted from the original image by computational analysis and stored to form an intermediate matrix. Subsequently, a series of texture features are computed based on this intermediate matrix. Texture features can be categorized into different types depending on the intermediate matrices used.

**Table 2:** Distribution of radiomic features.

	Feature	N
	Total	1246
Original	First order features	18
Original	2D/3D Shape features	14
Original	GLCM based features	24
Original	GLRLM based features	16
Original	GLSZM based features	16
Original	GLDM based features	14
Filtered	LOG based features	440
Filtered	Wavelet based features	704

GLCM, Gray Level Co-occurrence Matrix; GLRLM, Gray Level Run Length Matrix; GLSZM, Gray Level Size Zone Matrix; GLDM, Gray Level Dependence Matrix.

In this study, the four most commonly used matrices for radiomics analysis of pulmonary nodule images were calculated: the gray level co-occurrence matrix (GLCM), the gray level run length matrix (GLRLM), the gray level size zone matrix (GLSZM), and the gray level dependence matrix (GLDM). Filtered features include first-order and texture features obtained after applying filters to modify the original image. The modified images are then reanalyzed to extract features. In this study, we applied two widely used filters for the radiomic analysis of pulmonary nodules: the wavelet and Laplacian-of-Gaussian (LOG) filters. Features derived from these transformations are referred to as Wavelet features and LOG features respectively. When the image is transformed without using filters, the first-order, shape, and texture features extracted directly from the original image are categorized as original features.

### 2.4 Statistical analysis

Statistical analyses were conducted using R language (R-4.0.1) and SPSS 25.0 (IBM, Armonk, NY, USA). Charts were created using GraphPad Prism 8 (GraphPad Software Inc., San Diego, CA, USA) and WPS Office 2022. Statistical significance was defined as P < 0.05.

#### 2.4.1 Consistency analysis

Intra- and interclass correlation coefficients (ICCs) were calculated to evaluate the reproducibility of diameter measurements. Thirty cases of pulmonary nodules were randomly selected, and measurements were performed as follows: Doctors A and B were invited to perform the diameter measurements. Doctor B repeated the diameter measurements for the same 30 cases after a 3-week interval. The ICCs of the diameter measurements were calculated to evaluate consistency. At P < 0.05, an ICC value > 0.75 represents a high degree of reproducibility.

#### 2.4.2 Comparison of clinical baseline data

The chi-square test was used for count data comparison. If the total sample size was <40 or the minimum theoretical frequency was <1, the Fisher exact probability test was applied. For measured data, a one-way analysis of variance was used when normality and homogeneity of variance were satisfied. Otherwise, the Kruskal–Wallis H test was applied. For correlation analysis, Pearson correlation analysis was used for continuous variables that followed a normal distribution. Otherwise, Spearman’s correlation was used for non-normally distributed variables. Bonferroni correction was applied for test-level adjustment.

#### 2.4.3 Segmented ROI coherence analysis

This study employed the DC to evaluate the variability of observer segmentation of ROI. The DC is a widely used metric for evaluating the similarity between two sets, particularly in image segmentation tasks. The DC ranges from 0 to 1, with values closer to 1 indicating greater similarity. The formula for calculating DC is as follows:


$$Dice(A,B)=\frac{2|A\cap B|}{\left|A|+|B\right|}$$

To compare the differences in segmentation ROIs between the six doctors and the AI algorithm, we selected the segmentation ROI of Doctor A, the most senior cardiothoracic diagnostician as the reference. The DC was calculated for the segmentation ROIs of the other five doctors and the AI algorithm was compared to Doctor A to enable between-group comparisons. To further explore the impact of segmentation ROI variations on radiomic features, the seven segmentation groups (six doctors and an AI algorithm) were paired for two-by-two comparisons. The median DC from these pairings was used as the final segmentation result for subsequent analysis.

#### 2.4.4 Stability analysis of radiomic features

The overall concordance correlation coefficient (OCCC) was employed to quantify the effect of IOV of segmentation ROIs on radiomic feature stability (30). The OCCC, suitable for assessing measurement consistency across large samples involving three or more observers, ranges from 0 to 1. Higher values indicate better inter-observer measurement consistency (30–32).

In this study, OCCC <0.5 indicates poor feature stability, significantly affected by segmentation ROI differences; 0.5 ≤ OCCC < 0.75 suggests average feature stability, moderately affected by segmentation ROI differences; 0.75 ≤ OCCC < 0.90 indicates good feature stability, minimally affected by segmentation ROI differences; OCCC ≥ 0.90 indicates very good feature stability, negligibly affected by segmentation ROI differences. If the OCCC of a feature is ≥0.75, the feature is considered to have good stability in this study.

## 3 Results

### 3.1 Observer Reliability

In this study, 30 randomly selected pulmonary nodular lesions were analyzed to validate the reproducibility of diameter measurements. The ICC value for measurements between Doctor A and Doctor B was 0.960 (95% CI: 0.919–0.981). After a 3-week interval, Doctor B repeated diameter measurements for the same 30 lesions, yielding an ICC value of 0.951 (95% CI: 0.899–0.976). These findings demonstrate that the diameter measurements in this study were highly reproducible and reliable.

### 3.2 Baseline and imaging data

Following rigorous screening, 88 patients with 232 nodular lesions were included in the study. Detailed clinical and imaging data for all nodular lesions are presented in **Table 3**. Among the 232 pulmonary nodular lesions, 81 (34.91%) were SN, 40 (17.24%) were pSN, and 111 (47.84%) were pGGN. The median age of the patients was 57 years (interquartile range [IQR]: 14 years), with the majority being female (68.97%). No statistically significant differences were observed in age distribution among the SN, pSN, and pGGN groups. The median ages for these groups were 61 years (IQR: 14 years), 54 years (IQR: 15 years), and 55 years (IQR: 11 years), respectively.

**Table 3:** Distribution of baseline and imaging data for all pulmonary nodules.

		Total	SN	pSN	pGGN	P-Value
		232	81 (34.91%)	40 (17.24%)	111 (47.84%)	
Gender	Male	72 (31.03%)	40 (49.38%)^a^	10 (25.00%)^b^	22 (19.82%)^b^	<0.001
	Female	160 (68.97%)	41 (50.62%)^a^	30 (75.00%)^b^	89 (80.18%)^b^	
Age (years)		57 (14)	61 (18)	54 (15)	55 (11)	0.135
Nodule size		0.56 (0.40)	0.49 (0.25)^a^	0.94 (0.78)^b^	0.54 (0.24)^a^	<0.001
Nodule location 1	the left-up lobe	58 (25.00%)	9 (11.11%)	10 (25.00%)	39 (35.14%)	–
	the left-down lobe	41 (17.67%)	24 (29.63%)	8 (20.00%)	9 (8.11%)	
	the right-up lobe	63 (27.16%)	14 (17.28%)	8 (20.00%)	41 (36.94%)	
	the right-middle lobe	12 (5.17%)	7 (8.64%)	2 (5.00%)	3 (2.70%)	
	the right-down lobe	58 (25.00%)	27 (33.33%)	12 (30.00%)	19 (17.12%)	
Nodule location 2	peripheral	230 (99.14%)	81 (100.00%)	39 (97.50%)	110 (99.10%)	0.437*
	centralized	2 (0.86%)	0 (0.00%)	1 (2.50%)	1 (0.90%)	

a, b show the results of inter-group comparisons: identical letters indicate no statistically significant difference, while different letters indicate a statistically significant difference. *Fisher’s exact probability method: Each subscript letter indicates a subset of the lesion type category, and the proportions of columns in these categories are not significantly different from each other at a level of 0.05. Count data were expressed as numbers of cases (percentages), and measures were expressed as medians (interquartile spacing) unless otherwise specified. pSN, partially solid nodule; pGGN, pure ground glass nodule.

The median diameter of all nodules was 0.56 cm (IQR: 0.40 cm) (**Table 3**). Notably, the median diameter of pSN was significantly larger than that of SN/pGGN (median, 0.94 cm vs. 0.49 cm/0.54 cm, respectively; P < 0.001). Regarding positional distribution, the nodules were located as follows: 58 (25.00%) in the left-up lobe, 41 (17.67%) in the left-down lobe, 63 (27.16%) in the right-up lobe, 58 (25.00%) in the right-down lobe, and 12 (5.17%) in the right-middle lobe. Of the total nodules, 99.14% (230 cases) were peripheral pulmonary nodules.

### 3.3 Segmented ROI coherence analysis

To evaluate the consistency of the segmentation ROIs between observers and the AI algorithm, the segmentation result of Doctor A, the cardiothoracic diagnostician, was selected as the reference. The DC for the segmentation ROIs of the other five doctors and the AI algorithm were calculated and analyzed for between-group comparisons. As summarized in **Table 4**, statistically significant differences were observed between the DC distributions of the other five doctors and the AI algorithm (p < 0.001). This trend was consistent across all nodules and within each subtype of nodules, when the DCs were calculated using the segmentation ROIs of Doctor A as a reference. Notably, the segmentation results of the AI algorithm were similar to those of the trained non-radiology clinician, Doctor F.

**Table 4:** Consistency analysis of ROIs for pulmonary nodules.

	Total	Solid nodule	Part solid nodule	pGGN
Doctor A	—	—	—	—
Doctor B	0.75 (0.13)	0.78 (0.11)	0.78 (0.16)	0.72 (0.14)
Doctor C	0.76 (0.12)	0.77 (0.13)	0.79 (0.11)	0.75 (0.10)
Doctor D	0.80 (0.11)	0.81 (0.12)	0.79 (0.10)	0.80 (0.10)
Doctor E	0.72 (0.16)	0.70 (0.19)	0.75 (0.14)	0.72 (0.16)
Doctor F	0.66 (0.18)	0.67 (0.17)	0.70 (0.22)	0.64 (0.15)
AI algorithm (automatic)	0.69 (0.17)	0.70 (0.16)	0.75 (0.16)	0.66 (0.17)
P-value	<0.001	<0.001	<0.001	<0.001

Count data were expressed as the number of cases (percentage), and measures were expressed as medians (interquartile spacing) unless otherwise specified.

pGGN, pure ground glass nodule.

**Figure 4:**
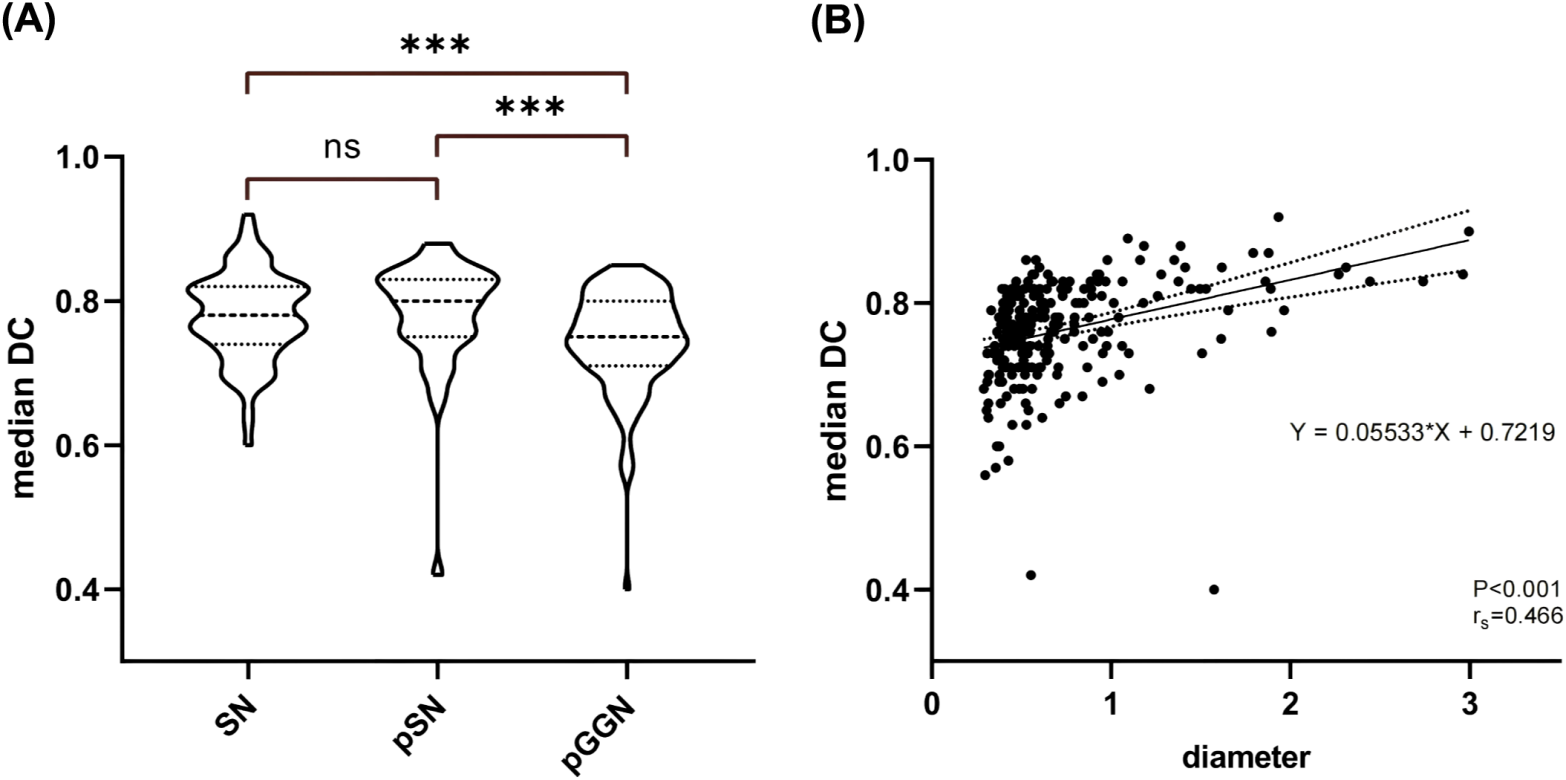
Nodal DC Analysis. **(A)** Comparison of median DC distribution between different types of nodules. **(B)** Results of correlation analysis and linear regression analysis between nodule diameter and median DC. SN, solid nodule; pSN, partially solid nodule; pGGN, pure ground glass nodule.

**Figure 4A** illustrates the variability in the median DC distributions across different pulmonary nodule types. The median DC values for pGGN (median: 0.75, IQR: 0.09) were significantly lower compared to SN (median: 0.78, IQR: 0.08) and pSN (median: 0.80, IQR: 0.08). A moderate positive correlation was identified between nodule diameter and the median DC (rs = 0.466, P <0.001). As shown in **Figure 4B**, the median DC increased by 0.055 for each unit increase in nodule diameter.

### 3.4 Stability analysis of radiomic features

In this study, we utilized the Pyradiomics package for radiomic feature extraction. A total of 1246 radiomic features were extracted from each nodule ROI, including 102 original features, 440 LOG features obtained from LOG-transformed images, and 704 wavelet features obtained from wavelet-transformed images. The original features included 18 original_first order features, 14 original_shape features, 24 original_GLCM features, 16 original_GLRLM features, 16 original_GLSZM features, and 14 original_ GLDM features.

#### 3.4.1 Stabilization rate of radiomic features

**Table 5** displays the OCCC distribution of the radiomic features. Of the total features, 1071 (85.96%) demonstrated good stability (OCCC ≥ 0.75), with 766 (61.48%) exhibiting very good stability (OCCC ≥ 0.90). Among the eight types of radiomic features, the original _first_order, original_GLCM, original_GLRLM, original_GLSZM, LOG, and wavelet features all had stable feature rates exceeding 80.00%. Notably, (91.59%) of the LOG features showed good stability. The shape and original_GLDM features both had stable feature rates of 78.57%.

**Table 5:** Distribution of radiomic features characteristics.

	Total	OCCC<0.5	0.5≤OCCC<0.75	0.75≤OCCC<0.90	OCCC≥0.90	
	Total	1246	28 (2.25%)	147 (11.80%)	305 (24.48%)	766 (61.48%)
Original	First order features	18	1 (5.56%)	2 (11.11%)	5 (27.78%)	10 (55.56%)
Original	2D/3D Shape features	14	1 (7.14%)	2 (14.29%)	1 (7.14%)	10 (71.43%)
Original	GLCM based features	24	1 (4.17%)	2 (8.33%)	5 (20.83%)	16 (66.67%)
Original	GLRLM based features	16	0 (0%)	3 (18.75%)	2 (12.50%)	11 (68.75%)
Original	GLSZM based features	16	0 (0%)	3 (18.75%)	4 (25.00%)	9 (56.25%)
Original	GLDM based features	14	1 (7.14%)	2 (14.29%)	1 (7.14%)	10 (71.43%)
Filtered	LOG based features	440	4 (0.91%)	33 (7.50%)	96 (21.82%)	307 (69.77%)
Filtered	Wavelet based features	704	20 (2.84%)	100 (14.20%)	191 (27.13%)	393 (55.82%)

GLCM, Gray Level Co-occurrence Matrix; GLRLM, Gray Level Run Length Matrix; GLSZM, Gray Level Size Zone Matrix; GLDM, Gray Level Dependence Matrix.

#### 3.4.2 Comparison of radiomic features OCCC

**Figure 5:**
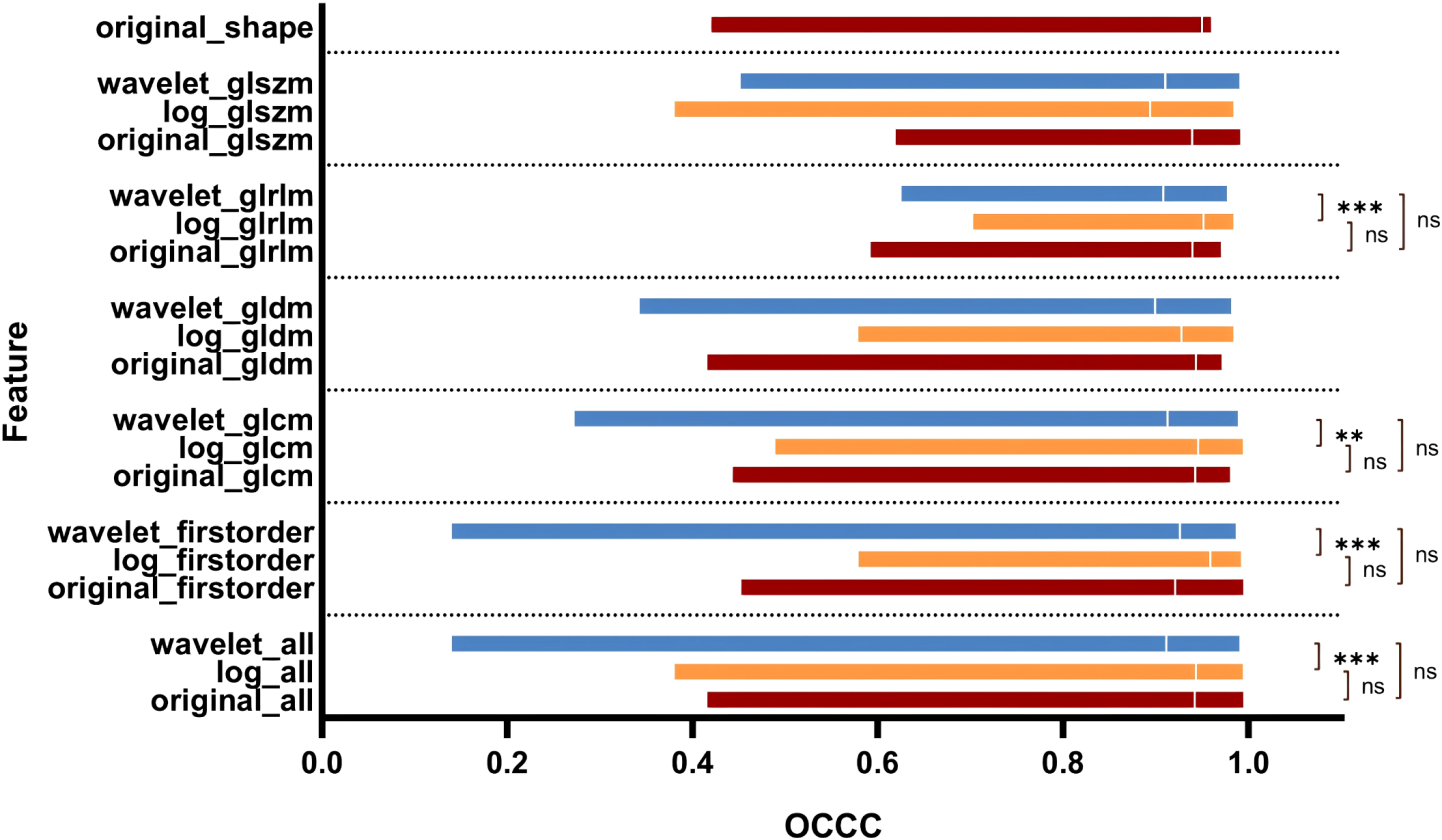
OCCC analysis of radiomic features of pulmonary nodules. GLCM, Gray Level Co-occurrence Matrix; GLRLM, Gray Level Run Length Matrix; GLSZM, Gray Level Size Zone Matrix; GLDM, Gray Level Dependence Matrix.

**Figure 5** illustrates the OCCC analysis results of the 1246 radiomic features. The original features exhibited good stability (median OCCC: 0.92–0.95, IQR: 0.12–0.19), both in the overall distribution and in the different feature categories. Overall, the median OCCC values for the LOG features (median: 0.94, IQR: 0.08) were significantly higher than those for the wavelet features (median: 0.91, IQR: 0.13). Specifically, the stability of the LOG features, particularly the first-order, GLCM, and GLRLM features was better than that of the wavelet transform (P < 0.05).

**Figure 6:**
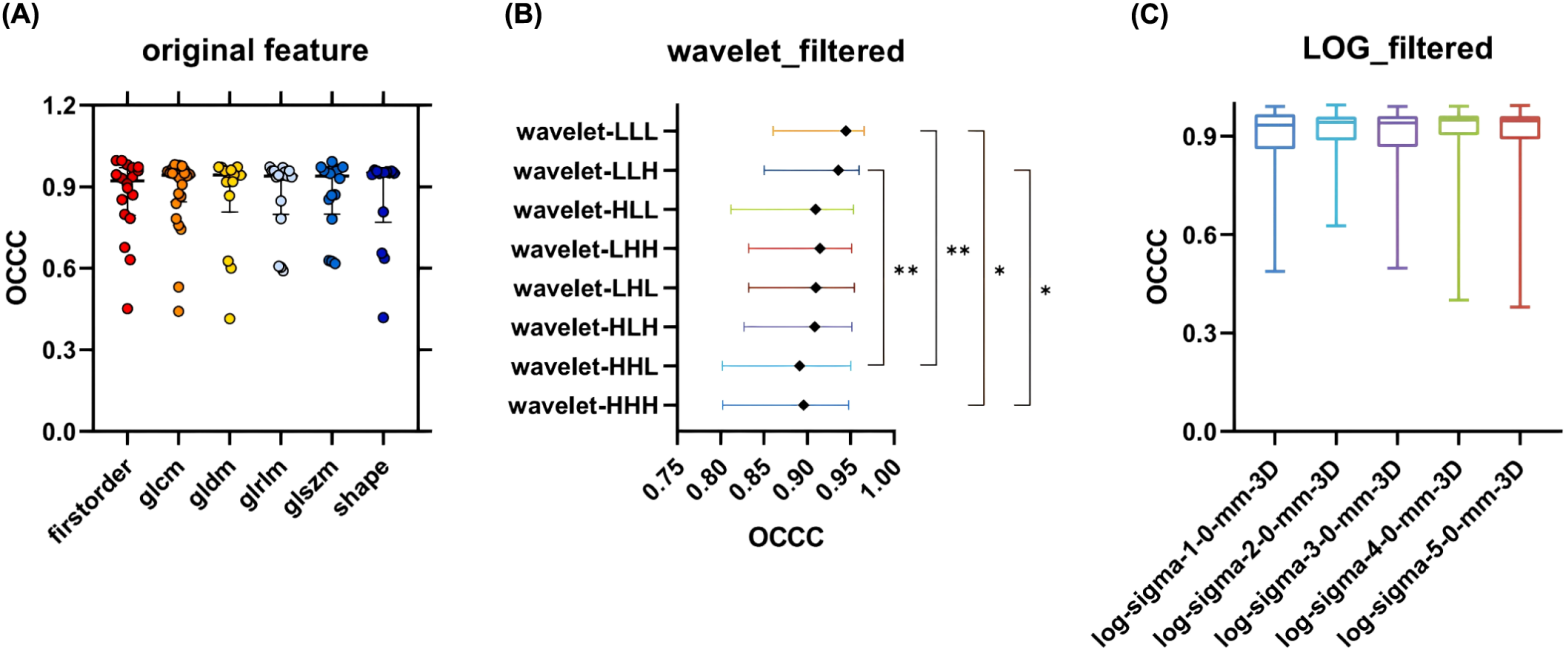
OCCC analysis of radiomic features of subgroups. **(A)** Comparison of OCCC distributions of 6 types of radiomic features in Original feature; **(B)** Comparison of OCCC distributions between groups of radiomic features obtained by 8 transformations in Wavelet feature; **(C)** Comparison of OCCC distributions between groups of radiomic features obtained by 5 transformations in LOG feature. GLCM, Gray Level Co-occurrence Matrix; GLRLM, Gray Level Run Length Matrix; GLSZM, Gray Level Size Zone Matrix; GLDM, Gray Level Dependence Matrix.

The median OCCC value of the Wavelet_GLSZM features (median: 0.91, IQR: 0.15) obtained after wavelet transformation image extraction was higher than that of LOG_GLSZM features (median: 0.89, IQR: 0.13); however, the difference was not statistically significant (P > 0.05). There was no statistically significant difference in stability between the subgroups of original and LOG features (P > 0.05). In contrast, there was a statistically significant difference in the distribution of feature stability for wavelet feature subgroups (P < 0.05) (**Figure 6**), with radiomic features extracted from the Wavelet_LLL and Wavelet_LLH transformation modalities showing relatively high stability (**Figure 6B**).

**Figure 7:**
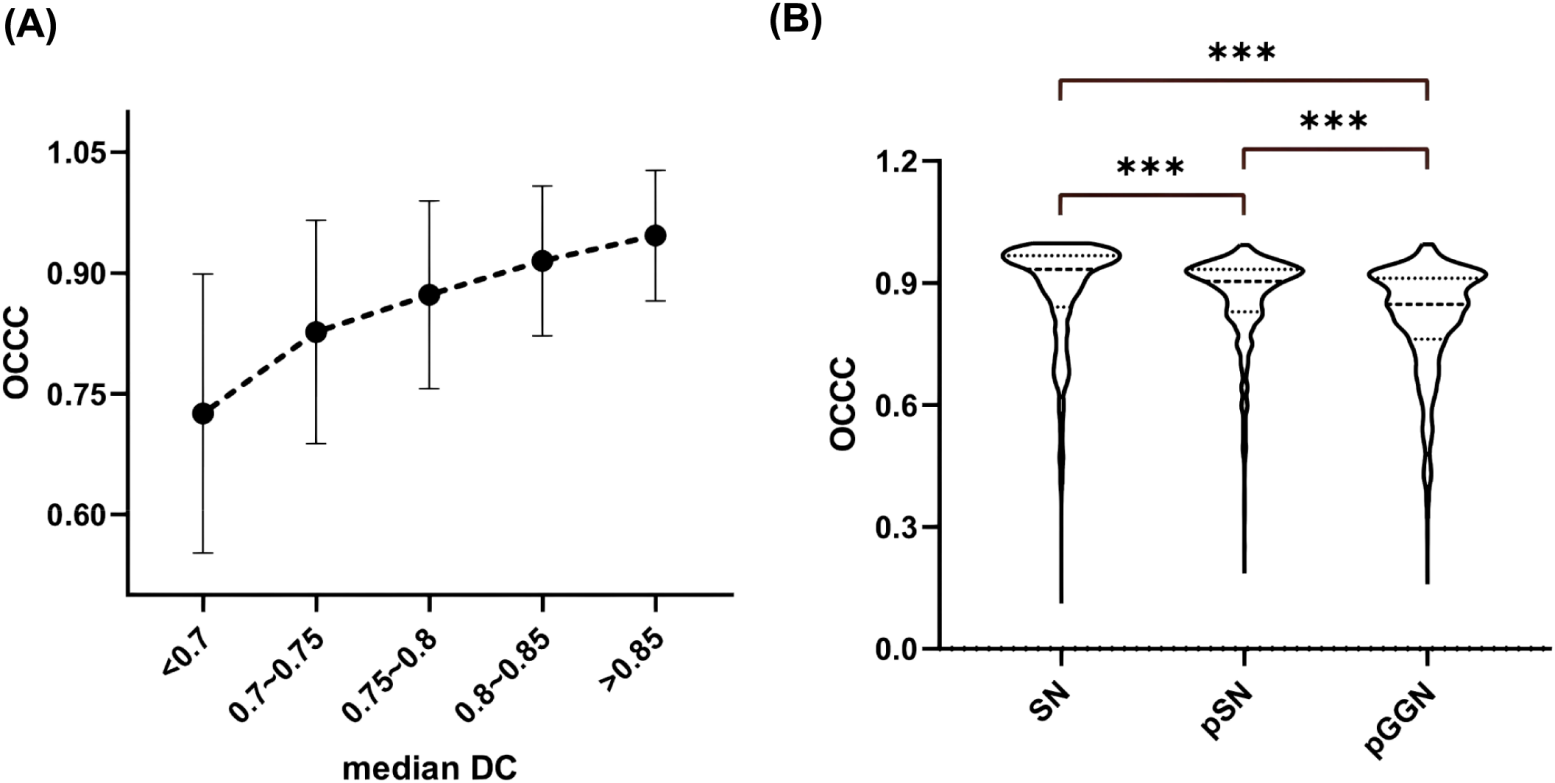
Nodal OCCC analysis. **(A)** Median DC versus OCCC distribution; **(B)** Comparison of OCCC distribution between different types of nodules. SN, solid nodule; pSN, partially solid nodule; pGGN, pure ground glass nodule.

The stability of the radiomic features varied among the different types of nodules (**Figure 7B**), with SN exhibiting the best stability (median OCCC: 0.93, IQR: 0.13), followed by pSN (median OCCC: 0.90, IQR: 0.10), and pGGN showing the lowest stability (median OCCC: 0.85, IQR. 0.15). A significant positive correlation was observed between median DC and characteristic OCCC values. As the median DC increased, the characteristic OCCC also increased gradually (**Figure 7A**).

## 4 Discussion

Data segmentation is a crucial aspect of radiomics research, as IOV can significantly influence the values of radiomic features, thereby affecting the outcomes of radiomics research (5, 19). While some studies have investigated the impact of outlining ROI variability on radiomic features, these have primarily focused on different types of tumor lesions with small samples, resulting in inconsistent and subjective conclusions. Moreover, although there are many studies on the radiomics of pulmonary nodules, few have specifically addressed the IOV analysis of the radiomic features and segmentation results of pulmonary nodules. This gap in the literature underscores the need for a systematic analysis of the radiomic characteristics of pulmonary nodules, exploring the influence of IOV on these features. Such an analysis could provide valuable reference information for selecting appropriate segmentation modalities in future radiomics studies. It would also contribute to the standardization of radiomics and facilitate the clinical application of radiomics-based techniques, offering significant clinical and scientific insights.

In this study, we included 232 pulmonary nodule lesions and enlisted six doctors with varying levels of experience and expertise in different medical specialties to outline the lesions. Additionally, we employed the modified 3D-U-Net for fully automated algorithmic segmentation of pulmonary nodule lesions, enabling a comprehensive and systematical evaluation of the impact of IOV in segmentation ROI on the radiomic features of pulmonary nodules.

### 4.1 Segmented ROI consistency

In a study by Leo et al, 11 radiologists with varying experience were tasked with outlining lesions, including liver cancer, lung cancer, intracranial hematomas, and renal contours. A total of 3,193 ROIs were collected and analyzed, revealing that the IOV in segmentation results was influenced by the type of lesion. The highest variability in ROI volume was observed in lung cancer lesions, with a 20.8% variation [-8.8, +10.2%] (17). Similarly, Matea et al. analyzed the DC values of segmentation results for NSCLC, head and neck squamous cell carcinoma, and malignant pleural mesothelioma. Their findings suggested a significant correlation between the stability of radiomic features and the median DC of the segmentation results, with variability in the stability of radiomic features (18). In alignment with previous studies, our findings demonstrated significant differences in the segmentation ROI consistency between doctors, both for all nodules and for lesions in different subgroups (SN, pSN, and pGGN). The results suggested that the median DC was significantly higher for SN and pSN compared to pGGN. The pGGN had a higher segmentation IOV that could be attributed to its distinctive characteristics, such as ground-glass-like changes and generally smaller diameter, which can blur the tumor-pulmonary interface, making it more challenging to assess. In contrast, PSN lesions typically have higher density and more distinctive boundaries between the lesion and normal lung tissue, making outlining relatively less difficult. Moreover, we found a moderately positive correlation between nodule diameter and median DC (rs=0.466, P <0.001), with median DC increasing by 0.055 for each unit increase in nodule diameter (**Figure 4B**). This suggests that larger-diameter pulmonary nodules are easier for doctors to assess and outline. The significantly large diameters of pSN lesions compared to pGGN in this study may explain the better segmentation ROI concordance observed in pSN lesions. Taken together, our results indicate that IOV during segmentation of pulmonary nodules is influenced by both nodule size and type, with smaller pGGNs being more difficult to segment and exhibiting greater IOV.

### 4.2 Stability analysis of radiomic features

Gargi et al. conducted a study using CT images from 20 patients with lung cancer to explore the relationship between IOV in segmentation results and the stability of NSCLC radiomic features (18). Their results showed that most of the radiomics features were well stabilized and were not significantly affected by IOV (33). Another study analyzed tumor information from different sites and found that 90% of the radiomic features exhibited good stability across 11 NSCLC lesions (18). Christoph et al. examined three different public datasets, including the Lung Image Database Consortium (LIDC-IDR): Kidney Tumor Segmentation Challenge and Liver Tumor Segmentation Challenge. A total of 92 radiomic features were extracted and analyzed, revealing that 85% of these radiomic features exhibited good stability with an ICC greater than 0.80 (19).

In this study, we analyzed 232 cases of pulmonary nodules and extracted 1246 radiomic features from each nodule ROI. The analysis of the degree of influence of IOV on the radiomic features revealed that 85.96% (1071/1246) of the features were well stabilized (OCCC ≥ 0.75) and not easily affected by segmentation IOV. Furthermore, 766 (61.48%) of these features exhibited very good stability (OCCC ≥ 0.90). Although the research subjects and the number of features with good stability differ from those in previous studies, the general conclusion remains consistent: most radiomic features are stable and less affected by IOV in segmentation results.

#### 4.2.1 Original features

Previous studies have analyzed the radiomic features of tumors across different sites and found that shape, first-order, and GLCM features had the best stability, with average ICC values of 0.93, 0.91, and 0.92, respectively (19). Another study showed that GLDM, GLRLM, and GLSZM features had the best stability and lower sensitivity to the IOV associated with lesions outlining in patients undergoing lung cancer radiotherapy (33).

In this study, Original_GLCM had the highest feature stabilization rate, 87.50% (21/24). Other original features, including original_First_order, original_Shape, original_GLRLM, original_GLSZM, and original_GLDM, all demonstrated feature stabilization rates ranging from approximately (78.57%–83.34%). Although the stabilization rates varied across feature types, the between-group feature stability analysis showed no statistically significant difference in the distribution of OCCC values among the original feature categories (**Figure 6A**). Overall, the original features extracted in this study exhibited good stability.

#### 4.2.2 LOG features and wavelet features

The introduction of filters in radiomics increased the number of features, subsequently boosting the number of stable features. Ruben et al. analyzed CT images of 46 patients with NSCLC and found that the stability of wavelet features was poor (34). In contrast, this study observed that both LOG and wavelet features demonstrated better stability, with feature stability rates of 91.59% (403/440) and 82.95% (584/704), respectively. However, when analyzing the OCCC values, the median OCCC values for the LOG features were higher than those of the wavelet features across the first-order, GLCM, GLDM, and GLRLM features (**Figure 5**).

Although the median OCCC values of the wavelet features were higher than those of the LOG features in the GLSZM category, the differences were not statistically significant (P > 0.05). Additionally, feature stability varied significantly among the eight transform subgroups of wavelet features, with the Wavelet_LLL and Wavelet_LLH features showing greater stability compared to the Wavelet_HHH and Wavelet_HHL features (P < 0.05). The distributions of OCCC values among the five transformed LOG features subgroups were similar (**Figure 6C**). In summary, the LOG features were better stabilized than the wavelet features in this study. The fluctuations in OCCC values among different wavelet transform subgroups suggest that an appropriate selection of wavelet features should be considered in future radiomic studies of pulmonary nodules.

### 4.3 DICE and OCCC

Gargi et al. found that a higher DC, which indicates greater consistency in segmentation results, correlates with a higher rate of radiomic feature stabilization (33). In this study, a positive relationship was observed between the median DC and the median OCCC of the pulmonary nodules, with both values increasing together. This finding is consistent with previous research. Additionally, this study observed that the stability of radiomic features varied across different types of nodules, with the highest OCCC values observed in SN and the lowest in pGGN. This may be attributed to the lower segmentation ROI consistency of pGGN, as discussed earlier, reaffirming the critical role of segmentation result consistency in radiomic feature stability.

Lesion segmentation is a crucial step in radiomics studies, and both previous studies and the present study have confirmed that improving the consistency of segmentation results can enhance the stabilization rate of radiomic features. Therefore, strategies to reduce IOV in segmentation results should be implemented to ensure the stability of radiomic features and improve the reproducibility of study findings. First, standardized guidelines for lesion segmentation should be formulated, and all personnel should receive training before performing segmentation. Clear and objective requirements can help reduce the influence of subjective biases. Second, post-processing techniques, such as algorithms designed to exclude unnecessary structures (bones, air, etc.), may further enhance segmentation consistency.

However, despite rigorous training and careful post-processing, manual segmentation is inevitably affected by subjectivity, with variations in work experience and expertise potentially leading to differences in how segmenters’ perceptions of lesions. The use of computerized segmentation algorithms has been shown to help improve the consistency of segmentation results (13, 35). Chintan et al. found that automated segmentation methods yielded an ICC value of 0.85 ± 0.15 for radiomic features, significantly higher than the ICC of manual segmenters (ICC=0.77 ± 0.17) (35). While algorithmic segmentation is objective and reproducible, its accuracy still lags behind that of manual segmentation, which remains a key concern regarding its clinical application. The AI algorithm constructed in this study has a relatively simple structure, but its segmentation results after training with the LIDC-IDRI dataset are comparable to those of trained clinicians, meeting the needs of this study. With ongoing improvements in algorithms and the expansion of training data, the efficiency and accuracy of computerized segmentation algorithms have steadily improved. Some AI software are now equipped with advanced automatic segmentation tools designed for pulmonary nodules (36–38).

Both previous and present studies have demonstrated that most radiomics features are stable, with the impact of segmentation IOV being relatively limited. Furthermore, improving the consistency of segmentation results further enhances the reproducibility of radiomics analysis. In conclusion, the use of computerized algorithms for lesion segmentation improves the efficiency of radiomic studies and also reduces the impact of IOV on radiomic features by providing relatively high objectivity and reproducibility in segmentation results. Therefore, computerized algorithms should be considered for ROI outlining in subsequent radiomic studies of pulmonary nodules, based on specific research requirements.

### 4.4 Research limitations

Firstly, this study included data from 232 pulmonary nodules, which, although larger than previous studies, remains relatively small compared to the number of radiomics features analyzed. Further expansion of the sample size and repeated validation experiments are necessary. Secondly, to minimize the influence of scanning parameters on the results, case images in this study were obtained from a single device. Future experimental validation using different devices is needed. Lastly, radiomics is influenced by many factors. This study focused solely on the effect of segmentation differences on the radiomics of pulmonary nodules, without analyzing other potential influencing factors, such as image reconstruction parameters. In the future, we will expand the sample size and collect relevant information for a more comprehensive analysis.

## 5 Conclusions

In this study, we investigated the effect of (IOV) in segmentation results on the radiomic features of pulmonary nodules and assessed the stability of these features. Our findings indicate that IOV in segmentation results influences the radiomic features of pulmonary nodule images; however, most (85.96%) features were well stabilized and minimally affected by IOV. Enhancing segmentation ROI consistency helps reduce the impact of IOV on the radiomic features of pulmonary nodules. We observed variability across different radiomic features, with original and LOG features showing good stability, while wavelet features were more susceptible to IOV influence and exhibited relatively lower stability.

## Data Availability

The datasets generated and/or analyzed during the current study are not publicly available due to protecting individual patient privacy but are available from the corresponding author upon reasonable request. Requests to access the datasets should be directed to Hongjie Hu, hongjiehu@zju.edu.cn.
